# Rapid discrimination between wild and cultivated *Ophiocordyceps sinensis* through comparative analysis of label-free SERS technique and mass spectrometry

**DOI:** 10.1016/j.crfs.2024.100820

**Published:** 2024-08-14

**Authors:** Qing-Hua Liu, Jia-Wei Tang, Zhang-Wen Ma, Yong-Xuan Hong, Quan Yuan, Jie Chen, Xin-Ru Wen, Yu-Rong Tang, Liang Wang

**Affiliations:** a State Key Laboratory of Quality Research in Chinese Medicines, Macau University of Science and Technology, Taipa, Macau SAR, China; b Laboratory Medicine, Guangdong Provincial People's Hospital (Guangdong Academy of Medical Sciences), Southern Medical University, Guangzhou, Guangdong Province, China; c School of Medical Informatics and Engineering, Xuzhou Medical University, Xuzhou, Jiangsu Province, China; d Department of Laboratory Medicine, Shengli Oilfield Central Hospital, Dongying, Shandong Province, China; e School of Agriculture and Food Sciences, The University of Queensland, Brisbane, Queensland, Australia; f Centre for Precision Health, School of Medical and Health Sciences, Edith Cowan University, Perth, Western Australia, Australia; g Division of Microbiology and Immunology, School of Biomedical Sciences, University of Western Australia, Crawley, Western Australia, Australia

**Keywords:** *Ophiocordyceps sinensis*, Cultivation, Surface-enhanced Raman spectroscopy, Machine learning, Metabolomics

## Abstract

*Ophiocordyceps sinensis* is a genus of ascomycete fungi that has been widely used as a valuable tonic or medicine. However, due to over-exploitation and the destruction of natural ecosystems, the shortage of wild *O. sinensis* resources has led to an increase in artificially cultivated *O. sinensis*. To rapidly and accurately identify the molecular differences between cultivated and wild *O. sinensis*, this study employs surface-enhanced Raman spectroscopy (SERS) combined with machine learning algorithms to distinguish the two *O. sinensis* categories. Specifically, we collected SERS spectra for wild and cultivated *O. sinensis* and validated the metabolic profiles of SERS spectra using Ultra-Performance Liquid Chromatography coupled with Orbitrap High-Resolution Mass Spectrometry (UPLC-Orbitrap-HRMS). Subsequently, we constructed machine learning classifiers to mine potential information from the spectral data, and the spectral feature importance map is determined through an optimized algorithm. The results indicate that the representative characteristic peaks in the SERS spectra are consistent with the metabolites identified through metabolomics analysis, confirming the feasibility of the SERS method. The optimized support vector machine (SVM) model achieved the most accurate and efficient capacity in discriminating between wild and cultivated *O. sinensis* (accuracy = 98.95%, 5-fold cross-validation = 98.38%, time = 0.89s). The spectral feature importance map revealed subtle compositional differences between wild and cultivated *O. sinensis*. Taken together, these results are expected to enable the application of SERS in the quality control of *O. sinensis* raw materials, providing a foundation for the efficient and rapid identification of their quality and origin.

## Highlights

• Detailed identification and comparison of characteristic peaks of *O. sinensis* SERS were conducted.
• Metabolomics used to verify the feasibility of SERS technology.
• The SVM is utilized for accurately distinguishing wild and cultivated *O. sinensis.*
• Interpretation analysis is utilized to uncover significant Raman shifts in the decision-making process of models.

## Graphical abstract

**Figure:**
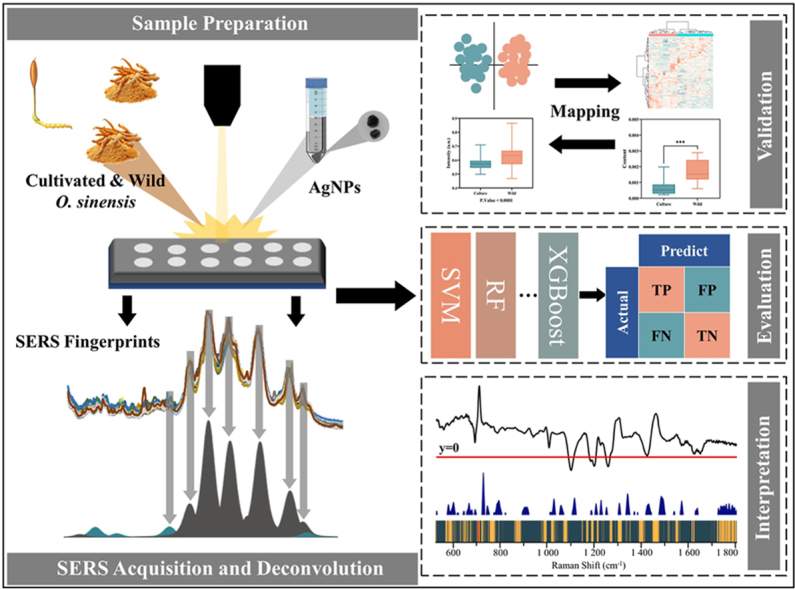

## 1 Introduction

*Ophiocordyceps sinensis* is a complex formed by a fungus parasitizing the larvae of special species of insects (Lepidoptera, Hepialidae) (Shrestha et al., 2010), and it has long been used as a traditional Chinese medicine (TCM) (Lo et al., 2013). Since 1980, many researchers have conducted extensive studies on *O. sinensis* and reported that it is rich in bioactive components, such as polysaccharides, nucleosides, sterols and amino acids, and other medicinal ingredients (Lo et al., 2013). At the same time, it has a variety of pharmacological activities, including hypoglycemic effects (C. Xu, Wu, Zou, Mao and Lin, 2023a), anti-tumor functions, and antioxidant activities (J. Xu et al., 2016). It can also enhance immune system capacity (Jang et al., 2015). Therefore, its highly regarded medicinal value has led to a significant increase in the market demand for *O. sinensis*. It is reported that *O. sinensis* is mainly distributed in the cold and high-altitude extreme environment of the Qinghai-Tibet Plateau in southwest China (Baral, 2017). Therefore, the unique host specificity and strict ecological environment conditions make the natural *O. sinensis* species very rare (Wei et al., 2021). To meet the growing demand, more and more artificially cultivated *O. sinensis* have emerged.

Previous studies have already shown that the composition of *O. sinensis* metabolites can be affected by the growing environment, which leads to significant differences in total amino acids, fatty acids, and mineral contents between cultivated and wild *O. sinensis* (Zuo et al., 2013). A Comparative examination between indoor-cultivated and wild *O. sinensis* demonstrated that their fatty acid composition shows a significant difference in the levels of polyunsaturated fatty acids (PUFAs). These observations and fatty acid data suggest that environmental factors, particularly temperature, soil pressure, and light intensity, strongly affect the growth of *O. sinensis* (Grosch et al., 2015). Another study shows that hypoxia plays an important role in the growth and synthesis of bioactive compounds in *Cordyceps militaris*. The introduction of *Vitreoscilla* hemoglobin enhanced the growth, biomass accumulation, and crude polysaccharides content of *Cordyceps militaris.* However, cordycepin production was decreased, and the yield of adenosine was increased significantly (Wang et al., 2021). Therefore, extensive studies have been conducted on the similarities and differences in pharmacologically active ingredients between wild and cultivated *O. sinensis* strains (Yue et al., 2013). Currently, many methods have been introduced to detect and quantify the components in *O. sinensis* to compare the compositional similarities and differences between cultivated and wild strains (L. Chen et al., 2018), (B. Zhang et al., 2020; He et al., 2023). Moreover, mass spectrometry-based metabolomics, as a powerful tool for identifying metabolites in organisms, has been widely used to compare the metabolites of *O. sinensis* from different geographical regions and cultivation conditions (J. Zhang et al., 2023). However, these methods involve expensive instruments, cumbersome pre-processing procedures, and specialized technicians. These issues highlight the urgent need to develop novel and convenient methods to effectively identify cultivated and wild *O. sinensis*.

To address the limitations of existing methods, various techniques have been developed to reduce the detection time and improve the detection accuracy of different types of *O. sinensis*, including UV spectroscopy (Tong et al., 2023), infrared spectroscopy (IR) (Du et al., 2017), nuclear magnetic resonance (NMR) (J. Zhang et al., 2015), and Raman spectroscopy (RS) (Wang et al., 2023c). Among them, RS is a chemical fingerprint detection method based on molecular vibration spectroscopy that captures information related to the vibration and conformation of biomolecules (Ma et al., 2023; Tang et al., 2024, Tang et al., 2024). Compared to other methods, the shorter wavelength excitation allows for higher spatial resolution, enabling the detection of smaller sample volumes (P. Wang et al., 2022). As an enhanced mode of RS, surface-enhanced Raman spectroscopy (SERS) is an ideal, nondestructive analytical approach for providing rich molecular fingerprinting information and intrinsic chemical characteristics. It is widely used in drug molecular detection and the detection of herbal medicines, as Yang et al. developed a platform for detecting plant chemicals and successfully compared the distribution of alkaloids in different parts of *Coptis chinensis*. They also evaluated the differences in content between different batches of *Coptis chinensis* decoction. (Yunpeng Wang et al., 2023; Yang et al., 2024a). In addition, SERS has been widely used in recent years for the detection of components in TCM and the analysis of both wild and cultivated biological samples (Yang et al., 2024b; You et al., 2017). For example, Xie et al. evaluated the presence of different active alkaloids in *Coptis chinensis* using SERS (Xie et al., 2024). Pećinar et al. compared the chemical compositions of cultivated and wild rosehips, discovering compositional differences in phenolics, polysaccharides, and lipids between the two rosehip types through RS (Pećinar et al., 2021). Although SERS is a powerful technique, detecting complex components via the analysis of raw spectral data is often interfered with complex random noises, peak overlaps, and subtle variations, which poses challenges for valuable information extraction and data interpretation (Tang et al., 2024, Tang et al., 2024). The emergence of signal processing methods based on chemometrics and machine learning (ML) has established an effective approach to overcome these challenges (Usman et al., 2023). For example, studies have successfully achieved accurate species classification and prediction by analyzing SERS spectra using ML models (H. Chen et al., 2021; J.-W. Tang et al., 2023). Farber et al. used orthogonal partial least squares discriminant analysis (OPLS-DA) to analyze the spectra of peanut leaves to discriminate between cultivated and wild peanut varieties (Payne and Kurouski, 2021). Therefore, SERS combined with ML algorithms can serve as a powerful strategy for distinguishing cultivated and wild species.

In this study, we first collected the SERS spectra generated from cultivated and wild *O. sinensis*. We then analyzed and deconstructed the characteristic peaks of SERS spectra to explore the biological significance behind these features. The metabolic profiles of wild and cultivated *O. sinensis* elucidated by SERS were also validated using Ultra-Performance Liquid Chromatography coupled with Orbitrap High-Resolution Mass Spectrometry (UPLC-Orbitrap-HRMS). Subsequently, we constructed various ML algorithms to analyze the spectral data and evaluate the performance of these algorithms through quantitative metrics. To explain the decision-making process of ML in identifying SERS spectral categories, we provided feature importance maps to elucidate the correlation between Raman patterns and *O. sinensis* components, thereby gaining insights into the differential components in cultivated and wild *O. sinensis*. The results showed that the ML algorithm Support Vector Machine (SVM) achieved the best performance and could quickly discriminate between wild and cultivated *O. sinensis* with 98.95% accuracy within 0.89 s. This demonstrated that the combination of the SERS technique with an interpretable ML model could effectively achieve the rapid and accurate identification of cultivated and wild *O. sinensis*, and the method had the potential to be extended to the study of other valuable TCM in similar situations.

## 2 Materials and methods

### 2.1 Chemicals and instruments

The wild *O. sinensis* samples (N = 12) were collected by and purchased from the local farmers at Changdu City, Tibet Autonomous Region, China. Cultivated *O. sinensis* (N = 15) were gifted by Dongguan East Sunshine Cordyceps Sinensis Research and Development Company (Dongguan, China), following the conventional culture procedures of the fungus (X.-W. Zhou, Li, & Tian, 2014). Both wild and cultivated *O. sinensis* were stored at 4 °C before the study. The chemical compounds used in this study include silver nitrate (AgNO_3_) and sodium citrate dihydrate (Na_3_C_6_H_5_O_7_), both of which were purchased from SinoPharm Chemical Reagent Co., Ltd (Shanghai, China). In addition, silicon wafers were purchased from Hangdan Optoelectronics Technology (China, Hangzhou). SERS signals of *O. sinensis* samples were detected by the Renishaw inVia™ confocal Raman microscope. The compositions of cultivated and wild *O. sinensis* samples were determined via the LC-MS system composed of Dionex UltiMate 3000 UHPLC system and LTQ Orbitrap Velos mass spectrometer (Thermo-Fisher Scientific, Germany).

### 2.2 Synthesis of silver nanoparticles (AgNPs)

The preparation process of silver nanoparticles was as previously described (Mou et al., 2024). Briefly, 33.72 mg silver nitrate was dissolved in 200 mL of deionized water and then the solution was heated to boiling on a magnetic stirrer (Huaxin Instrument Co. Ltd, Tianjin, China). Followed by adding 8 mL of 1% trisodium citrate dihydrate solution and stirring at 650 r/min to continue heating for 40 min. The reaction was carried out in the dark, and finally, heating was stopped until the solution was cooled to room temperature and sealed for storage. 1 mL of the solution was transferred and centrifuged at 7000 rpm for 7 min, and the precipitation was dissolved in 100 μL of deionized water to obtain a gray nano-silver solution. Supplementary Fig. S1 showed the morphology of silver nanoparticles, which was observed using transmission electron microscopy, and the particle size distribution of the material was analyzed. The UV–visible absorption spectrum showed that the absorption peak of AgNPs was located at 426 nm, which was consistent with previous reports (Lyu et al., 2023).

### 2.3 Sample preparation

Each whole *O. sinensis* plant was cut and ground and then passed through an 80-mesh sieve to obtain a fine powder sample. Weigh 4.0 mg of *O. sinensis* powder, put it in 40 μL deionized water at room temperature, and mix it using a vortex. After centrifugation in a microcentrifuge for 1 min, 10 μL of the supernatant was transferred and further mixed with 10 μL of the above-prepared silver nanoparticle solution. Therefore, a total of 20 μL of the mixture was spotted on a silicon wafer and waited for natural air drying before SERS signal detection. For metabolite analysis, *O. sinensis* powder was dissolved in deionized water (1:20, w/v) and then extracted by ultrasonication for 30 min. The mixture was then centrifuged at 14,000 g for 20 min and the supernatant was pushed through a 0.22 μm filter (Millipore, Merck) for analysis by PGC-LTQ/Orbitrap-HRMS.

### 2.4 SERS measurement and preprocessing

The Renishaw inVia™ confocal Raman microscope was used to collect SERS spectral data of *O. sinensis* samples, and 100 locations of dry samples on the silicon wafer were randomly selected for detection. The parameter settings of the Raman instrument include the excitation light wavelength of 785 nm, the Raman shift detection range of 500–1800 cm^−1^, the detection exposure time of 10s, and the laser power set at 0.1%. Before spectrum acquisition, the SERS characteristic peaks of the silicon wafer were used for spectrum calibration. All acquired SERS data were processed using the Unscrambler® X10.4 (CAMO, Oslo, Norway) for unit vector normalization, aiming to alleviate the influence of sample distribution and dimensional differences on data analysis.

### 2.5 SERS spectral analysis

The average SERS spectra were used to demonstrate the true distribution of the data and the standard deviation (SD) of each Raman shift was calculated to measure the consistency of the spectra (shaded region). Due to the high similarity between the SERS spectra of the two types of *O. sinensis*, the deconvolution method was employed to identify the distribution of characteristic peaks and to deconstruct the components of each SERS metabolic profile. All peak fittings were conducted using Origin software (2021, OriginLab, USA), with the *fit peaks pro* function utilized for fitting characteristic peaks, and the *Vogit* function used to extract detailed information on each spectral characteristic peak. Principal Component Analysis (PCA) was used to identify the main patterns in the SERS signals of *O. sinensis* and to explain the differences between the two groups of SERS metabolic profiles through PCA loadings. Two principal components, PC1 and PC2, were selected to capture the characteristics of the SERS signals. Specifically, the *fit_transform* operation was performed on the data using the *PCA* function from the *scikit-learn* library (version 0.21.3). The loadings generated by the *pca.components_* function were used to analyze the importance of characteristic peaks in distinguishing between different samples, and the *n_component* parameter is set to 2.

### 2.6 UPLC-Orbitrap-HRMS analysis

MS data were acquired using a UPLC-Orbitrap-HRMS equipped with electrospray ionization (ESI) in both negative and positive ionization modes. Spray voltage, 4 kV (±4 kV in ESI ±); Sheath gas (N2, >95%), 35 bar; Auxiliary gas (N2, >95%), 15 bar; heater temperature, 275 °C; Capillary temperature, 275 °C. The sample injection volume was 10 μL, and the components were separated on a HyperCard column (100 × 2.1 mm, 3 μm) (Thermo Fisher Scientific, Waltham, MA, USA). 5 mM ammonium acetate (Phase A) and acetonitrile (Phase B) were used as mobile phases. The total separation time was 25 min and the gradient program was set as follows: 0–2 min, 100% A, 2–5 min, 100-80% A, 5–10 min, 80-65% A, 10–15 min, 65-60% A, 15–20 min, 60-30% A; equilibration time of 5 min at 100% A. Column temperature: 35 °C. All system control, data acquisition, mass spectrometry data evaluation, and data processing were performed using XCalibur software version 2.0.7. The MS scan mode was a full MS scan from 50 m/z to 1500 m/z with a resolution of 35000. In ddMS2 mode, the normalized collision energy (NCE) was set to 35 with a resolution of 17500. The ion fragment assignments were obtained by matching high-precision quasi-molecular ions, isotopic distributions, and fragmentation patterns with the Reaxys database, the Natural Products Dictionary, and literature data.

### 2.7 Identification of metabolites

The GraphPad Prism software (GraphPad Software, USA) was employed to analyze the differences in metabolites identified through SERS peaks and metabolomics analysis. *Normality and lognormality tests* were performed to ascertain whether the data conformed to a normal distribution before conducting *t-tests*. For data meeting the normal distribution criteria, parametric tests were applied, and the homogeneity of variances was verified using the *Brown-Forsythe and Welch ANOVA tests*. Non-normally distributed data were analyzed by non-parametric testing using the *Mann-Whitney test Compare ranks* method for analysis. Visualization of SERS peaks and metabolite data from metabolomics was presented using box plots. The feasibility of the SERS technique was validated by comparing the trends in metabolites identified from SERS peaks with those from metabolomics analysis. The relationship between characteristic peaks was examined by employing the *corrplot* package in RStudio (version 2023.12.0 + 369) to compute the correlation coefficients among them. To reveal the relationships and differences among samples, PCA and OPLS-DA were employed to visualize the spatial distribution of SERS and metabolomic data. Clustering heatmaps were also utilized to analyze the differences in two types of *O. sinensis* metabolites. During the analysis process, SIMCA (version 13.0, 32-bit) was utilized for the analysis of SERS data, while MetaboAnalyst 6.0 (https://www.metaboanalyst.ca/) was employed for the analysis of metabolomic data.

### 2.8 Construction and evaluation of machine learning models

This study selected six commonly used ML algorithms, including Adaptive Boosting (AdaBoost), Decision Tree (DT), Gradient Boosting (GBoost), Random Forest (RF), SVM, and eXtreme Gradient Boosting (XGBoost), aiming to build optimal models for identifying cultivated and wild *O. sinensis*. Specifically, the input matrix includes two types of SERS, cultivated and wild, with a matrix shape of (2700, 1161). The *train_test_split* function was employed to split the dataset, with 70% of the data used for model training and 30% for testing. As the performance and generalization ability of ML models are directly related to hyperparameters, before model training, the hyperparameter ranges for each model were preset, ensuring that each set of optimal hyperparameter combinations falls within the given parameter intervals. The selection and range of hyperparameters for all models were provided in Supplementary Table S1. The *GridsearchCV* function was utilized to compute the accuracy of each parameter combination, combined with 5-fold cross-validation (cross_val_score, cv = 5), to ensure the stability and reliability of the selected models across various data partitions. The hyperparameter combination with the highest accuracy for each model was inputted into the function, and the fit method was executed to *fit* the model. All models use five evaluation metrics to comprehensively evaluate the performance of the model, namely Accuracy (*accuracy_score*), Precision (*precision_score*), Recall (*recall_score*), F1-score (*f1_score*), and Times (*datetime*). Among them, Precision and Recall are a set of complementary metrics, that were particularly effective in evaluating data imbalances (Wang et al., 2022, Wang et al., 2022). Additionally, three evaluation methods, 5-fold cross-validation (*cross_val_score*), Receiver Operating Characteristic (ROC) curve (*roc_curve, roc_auc_score*), and Confusion Matrix (*confusion_matrix*) were employed to assist in understanding the predictive results of the models. Algorithm analysis, evaluation metrics, and methods were implemented by calling functions in a scikit-learn library (version 0.21.3). The software and hardware versions used for data analysis were as follows: Python 3.8.18, Keras 2.10.0, numpy 1.24.4, matplotlib 3.7.2, tensorflow 2.10.0, pandas 2.0.3, CPU 19-13900HX, GPU RTX 4060.

### 2.9 Model interpretive analysis and feature importance

After comprehensively evaluating the performance of multiple classifiers, we conducted an interpretative analysis of the best machine-learning algorithm. In this study, SVM served as an effective and reliable choice that can effectively analyze the *O. sinensis* SERS data with the characteristics of "high dimensionality and small sample size". We utilized a "linear" SVM to find the optimal decision boundary between the two classes. The sign of the feature weights obtained from this algorithm indicates the direction of the feature prediction class (Wang et al., 2022, Wang et al., 2022, Wang et al., 2022). Therefore, we plotted the spectral feature importance map using the feature weights calculated by SVM (weight >0) and ranked the importance of the spectral feature peaks based on the distribution of the weight values. Subsequently, we compared the distribution of the feature peaks focused by SVM with the distribution of the original spectral feature peaks, further proving the feasibility of this method in distinguishing *O. sinensis* SERS data.

## 3 Results

### 3.1 Workflow for identifying cultivated and wild *O. sinensis*

**Fig. 1:**
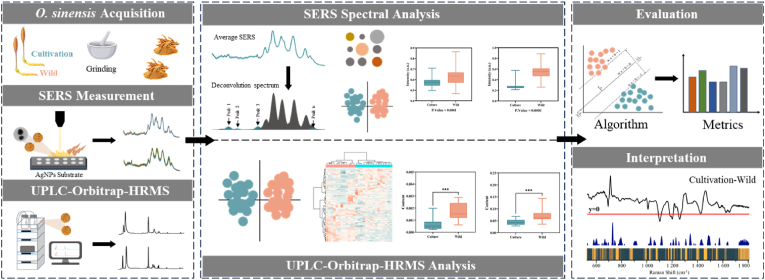
Workflow of classification and prediction of wild and cultivated *O. sinensis* samples based on SERS technology and ML algorithms.

The detection process for cultivated and wild *O. sinensis* is illustrated in Fig. 1. First, cultivated and wild *O. sinensis* were purchased and collected, then ground into powder. Subsequently, the chemical components in both types of *O. sinensis* samples were analyzed and compared using SERS technology and the UPLC-Orbitrap-HRMS platform, aiming to verify the feasibility of the SERS method. Following this, the SERS signals were input into various ML algorithms and comprehensively analyzed using different evaluation metrics to obtain the optimal discrimination model. Finally, to gain a deeper understanding of the decision-making process of the model, interpretive analysis was conducted on the algorithm with the best performance, identifying the key characteristic peaks involved in the model analysis process.

### 3.2 SERS spectral analysis of cultivated and wild *O. sinensis*

**Fig. 2:**
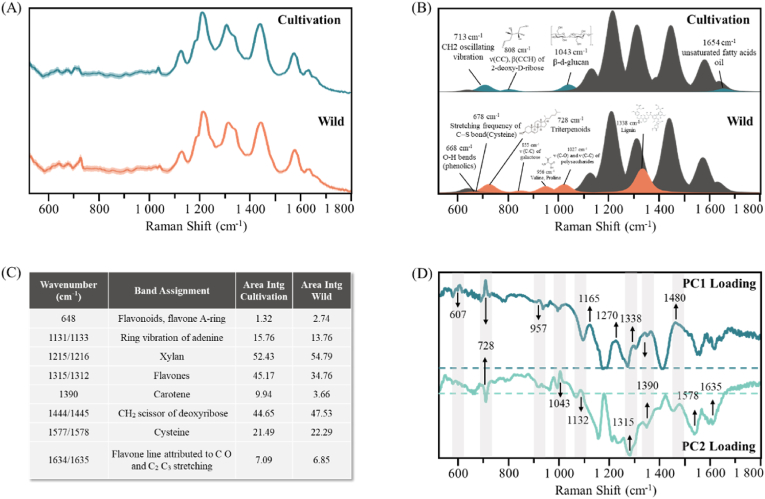
SERS spectral characteristic peaks and metabolite analysis. (A) Average SERS spectra of cultivated (N = 1500) and wild (N = 1200) *O. sinensis*, gray regions represent standard errors of SERS spectra within groups. (B) Deconvoluted SERS spectra of cultivated and wild *O. sinensis*, the highlighted color represents differential characteristic peaks, the dark gray indicates common characteristic peaks. (C) Common characteristic peaks and peak areas. (D) Loading plots of PC1 and PC2. (For interpretation of the references to color in this figure legend, the reader is referred to the Web version of this article.)

In this study, we calculated the average SERS signals of two types of *O. sinensis* (Fig. 2A). The distribution of spectral features indicates that the SERS signals of the two samples are similar. Therefore, we employed a deconvolution method to deconstruct the SERS signals. The highlighted regions in Fig. 2B (dark green and orange) demonstrate the unique characteristic peaks for each type of SERS. The Raman shifts of these characteristic peaks and their corresponding biological significance are provided in the figure. The dark gray area represents shared characteristic peaks. For these shared SERS peaks, we calculated the area for each peak (Fig. 2C). Significant differences were observed at the characteristic peaks located at 1315 and 1390 cm^−1^. The characteristic peak at 1315 cm^−1^ is attributed to flavones, a substance known for its antioxidant activity in *O. sinensis*, playing an important role in the human metabolic system (Q. Xu et al., 2018). The SERS peak at 1390 cm^−1^, attributed to the vibrations produced by carotenoids, has been confirmed to exist in *O. sinensis* and various fungal fruiting bodies (Jędrejko et al., 2021). Furthermore, by evaluating the loading plots of PC 1 and PC 2 (Fig. 2D), key spectra distinguishing cultivated from wild *O. sinensis* were preliminarily identified. The primary contributing scores for PC 1 were at 728, 957, 1338, 1390, and 1450 cm^−1^, with these characteristic peaks also appearing in the deconvolution-fitted spectral bands. Similarly, the key peaks in PC 2 for identifying different types of spectra were at 1043, 1132, 1315, 1578, and 1635 cm^−1^. In addition to the peaks identified by the deconvolution method, the four characteristic peaks at 607, 1165,1270 and 1480 cm^−1^ were also recognized by PCA as critical factors for differentiating the SERS of the two types of *O. sinensis*, indicating that ML methods do not rely solely on prominent spectra to distinguish *O. sinensis* categories. Supplementary Table S2 presents the peak positions of the main Raman vibrational modes reported in the literature and their corresponding compounds.

### 3.3 UPLC-Orbitrap-HRMS and SERS profiles analysis of *O. sinensis*

**Fig. 3:**
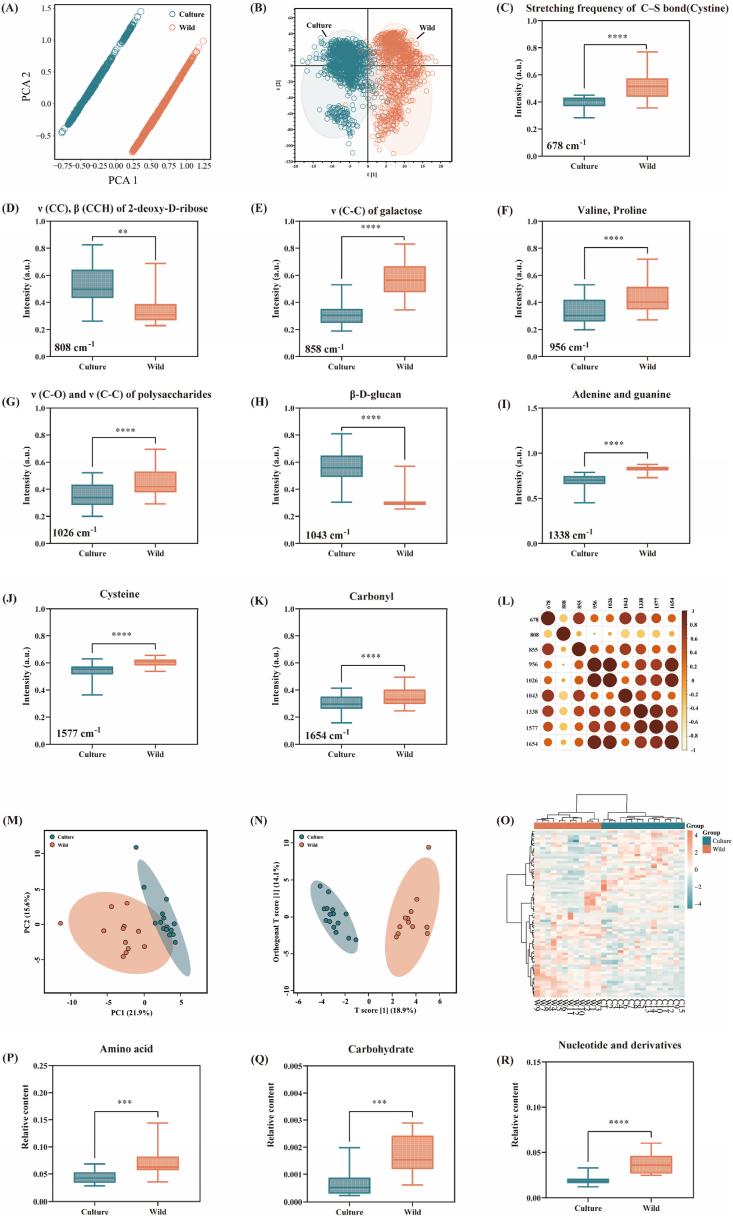
SERS and metabolomics profiles of wild and cultivated *O. sinensis*. (A) PCA cluster analysis, and (B) OPLS-DA cluster analysis of SERS spectra. (C–K) Box plot of major SERS peak intensities for cultivated (green) and wild (orange) *O. sinensis*. (L) Correlation analysis of significant SERS peaks. (M) PCA cluster analysis, and (N) OPLS-DA cluster analysis of metabolites discovered by metabolomics. (O) Heatmap analysis for metabolites in cultivated and wild groups. (P–R) Box plot of metabolites distinguishing among cultivated and wild groups. (For interpretation of the references to color in this figure legend, the reader is referred to the Web version of this article.)

In this study, we used PCA to analyze the cluster distribution of SERS spectral signals of cultivated and wild *O. sinensis* samples. The results showed that the samples in the same group clustered together and the two groups were well separated (Fig. 3A). OPLS-DA analysis showed that although there was a small overlap of spectral points in the SERS data, most of the spectral points had good separation (Fig. 3B). Therefore, Raman spectroscopy can effectively obtain the compositional information of cultivated and wild *O. sinensis* and distinguish between them. Fig. 3C–K shows 9 SERS peaks with significant differences in SERS intensity. Specifically, the SERS peak intensity of wild *O. sinensis* at 678 cm^−1^, 956 cm^−1^ and 1577 cm^−1^ is greater than that of cultivated *O. sinensis*. Among them, the SERS peaks at 678 cm^−1^ and 956 cm^−1^ are characteristic peaks that only exist in wild *O. sinensis* and are generated by the stretching frequency of C-S bond in cystine (Zhu et al., 2011) and the stretching vibration of the C-C bond in valine and proline (Fan et al., 2024), respectively. In addition, the peak at 1577 cm^−1^ comes from cysteine. Proline has important biological significance in plants exposed to various stress conditions, playing a favorable role in maintaining intracellular redox balance and energy status metabolism (Hayat et al., 2012). Valine, cysteine and cystine play important roles in regulating energy supply and immune function. (Scalise et al., 2016). This indicates that wild *O. sinensis* may have higher nutritional value.

Carbohydrates, composed of free sugars, polymerized sugars and sugar alcohols, are a group of essential ingredients with biological activities in *O. sinensis* (Yan et al., 2014). Previous studies have shown that the polysaccharides in *O. sinensis* have anti-oxidation, anti-tumor, hypoglycemic, and hypocholesterolemia biological activities, which might be used as markers for the quality control of *O. sinensis* (Koh et al., 2003; Ohta et al., 2007). In this study, the SERS peaks at 808 cm^−1^, 855 cm^−1^, 1026 cm^−1^, and 1043 cm^−1^ obtained by SERS detection belong to the vibration response of chemical bonds and groups in carbohydrates. In particularly, 808 cm^−1^and 1043 cm^−1^ are characteristic peaks in the cultivated *O. sinensis* samples, which are attributed to the stretching vibration of the C-C bond and in-plane bending vibration of the C-C-H bond of 2-deoxy-D-ribose, and β-D-glucan, respectively (Synytsya et al., 2023; Wiercigroch et al., 2017). Although the attribution of individual characteristic peaks in SERS detection cannot determine the specific compounds, the results show that there are differences in the content of carbohydrate components in wild and cultivated *O. sinensis*. Among them, the level of β-D-glucan is higher in cultivated *O. sinensis*, which may be produced by the extracellular polysaccharides of the cultured mycelium (Chatnarin and Thirabunyanon, 2023). Moreover, 855 cm^−1^ and 1026 cm^−1^ are characteristic peaks in the wild *O. sinensis*, which are attributed to the stretching vibration of the C-C bond of galactose ribose and the stretching vibration of the C-O bond and C-C bond of the polysaccharide, respectively(Marques et al., 2021; Souza et al., 2012). Studies have shown that the polysaccharides in wild *O. sinensis* are composed of glucose, galactose and mannitol (Tan et al., 2023). Therefore, SERS detected the signal of the peaks in 855 cm^−1^ and 1026 cm^−1^ in the wild *O. sinensis*, indicating that the polysaccharides content in the wild *O. sinensis* is higher than that in the cultivated *O. sinensis*.

Nucleosides are the principal component in *O. sinensis*, playing a key role in regulating various physiological processes, and mainly exhibit effective anti-viral, anti-inflammatory, antioxidant activities and neuroprotective functions(Y. Liu et al., 2015). Furthermore, it plays an important role in DNA/RNA synthesis and energy metabolism pathways (Ljungdahl and Daignan-Fornier, 2012). At present, more than 10 nucleosides (including adenosine, adenine, cytosine, cytidine, uridine, guanine, inosine, hypoxanthine, thymine, thymidine and 2′-deoxyuridine) have been isolated and identified from *O. sinensis* through various analysis methods (Y. Li et al., 2019; C. Xu, Wu, Zou, Mao and Lin, 2023b). A study compared the metabolite levels in wild and cultivated *O. sinensis* through metabolomics and transcriptomics analysis, revealing that the content of nucleotides and nucleosides derivatives in the wild *O. sinensis* is higher than that in the cultivated *O. sinensis* (J. Zhang et al., 2023). In this study, the vibration mode at 1338 cm^−1^ was only found in wild *O. sinensis* and was attributed to the vibration of adenine and guanine (Vaverkova et al., 2014), indicating that the content of nucleoside components in wild *O. sinensis* is higher. The accumulation of nucleosides is essential for the growth and survival of fungi, so the harsh growth environment of wild *O. sinensis* may lead to the elevated content of nucleosides. (Y. F. Xu et al., 2013). SERS detection revealed the peak at 1654 cm^−1^ is characteristic peaks of cultivated *O. sinensis*, generated by carbonyl groups in compounds. Furthermore, we used correlation analysis to understand the relationship between samples and metabolites, and the results showed that SERS spectra of metabolites from the same group and category were significant correlation (Fig. 3L). Therefore, SERS analysis revealed differences in the composition of multiple compounds between wild and cultivated *O. sinensis*, including carbohydrates, amino acids, and nucleosides.

Next, we used LC-MS to detect the metabolite components in wild and cultivated *O. sinensis*. The PCA clustering (Fig. 3M) and OPLS-DA analysis (Fig. 3N) of metabolites showed the differences in the expression levels of metabolites in wild and cultivated *O. sinensis*, respectively, and good separation was achieved between the groups. The differential metabolites evaluated by MS metabolomics were displayed by a heatmap (Fig. 3O), reflecting the expression trend of these metabolites. To fully understand the information of differential metabolites between wild and cultivated *O. sinensis*, we enriched and classified the compounds. The results showed significant differences in the composition of amino acids, carbohydrates and nucleic acids between the two groups (Fig. 3P–R), indicating that these metabolites are markers for distinguishing wild and cultivated *O. sinensis*. MS detected that the content of amino acid compounds such as valine, proline, leucine, histidine, and tyrosine was higher in wild *O. sinensis*, which was consistent with the enhancement of SERS characteristic peaks (678 cm^−1^, 956 cm^−1^ and 1577 cm^−1^). Note that MS can identify more molecular information than Raman spectroscopy, which may be related to the principles of the two detection methods. SERS detection generates response signals based on inelastic scattering caused by the vibration mode of the substance. However, MS uses the molecular mass-to-charge ratio (m/z) to identify chemical and structural information and has high specificity in distinguishing and identifying metabolites.

At the same time, the metabolite analysis results showed that carbohydrate components such as Rhamnose and 2-deoxy-D-galactose were abundant in wild *O. sinensis* samples, while carbohydrate components such as hexose and mannitol were higher in cultivated *O. sinensis*. The SERS analysis showed an enhancement of the SERS peaks at 855 cm^−1^ and 1026 cm^−1^, indicating that the content of galactose and glucan increased in wild *O. sinensis*, while the SERS peak intensity at the polysaccharide components (808 cm^−1^ and 1043 cm^−1^) was stronger in cultivated *O. sinensis*. It was revealed that mass spectrometry and SERS analysis are highly complementary, and the comprehensive analysis provides more comprehensive metabolic information to clarify the rich differential metabolite information in wild and cultivated *O. sinensis*. In addition, the peak intensity of adenine and guanine (1338 cm^−1^) is higher in wild *O. sinensis*, corresponding to the increasing trend of the metabolome. Therefore, SERS has great potential in distinguishing and identifying wild and cultivated *O. sinensis*.

### 3.4 Machine learning comparison and classification

To construct the optimal classifier for distinguishing between cultivated and wild *O. sinensis*, various evaluation metrics and methods were employed to comprehensively analyze each algorithm. The results (Table 1) indicate that SVM, due to its ability to efficiently handle high-dimensional and small sample data (Zhang et al., 2023, Zhang et al., 2023), achieved the best classification performance within 0.25s, with an accuracy of 97.99% and a 5-fold cross-validation score of 96.20%. Similarly, GBoost also attained comparable performance (accuracy = 97.87%), albeit requiring substantial computational resources for fitting. The identification accuracies of XGBoost, RF, and DT all exceeded 85%, suggesting that these three algorithms could serve as alternative strategies for distinguishing different types of *O. sinensis*. However, due to the lack of effective feature selection and the highly nonlinear distribution of *O. sinensis* SERS data, AdaBoost was unable to effectively capture the complex patterns within the data, resulting in an identification accuracy of only 73.23%.

**Table 1:** A comprehensive performance comparison among the six supervised ML algorithms.

Algorithm	Accuracy	Precision	Recall	F1	Time	5Fold
SVM	97.99%	97.99%	97.85%	97.99%	0.25s	96.20%
GBoost	97.87%	97.87%	97.91%	97.87%	232.98s	94.30%
XGBoost	97.47%	97.47%	97.72%	97.46%	0.33s	94.56%
RF	96.95%	96.95%	96.42%	96.95%	3.04s	94.30%
DT	85.35%	85.35%	85.82%	85.35%	0.03s	86.84%
AdaBoost	73.23%	73.23%	71.56%	72.99%	8.4s	64.18%

### 3.5 Evaluation and interpretation of machine learning models

**Fig. 4:**
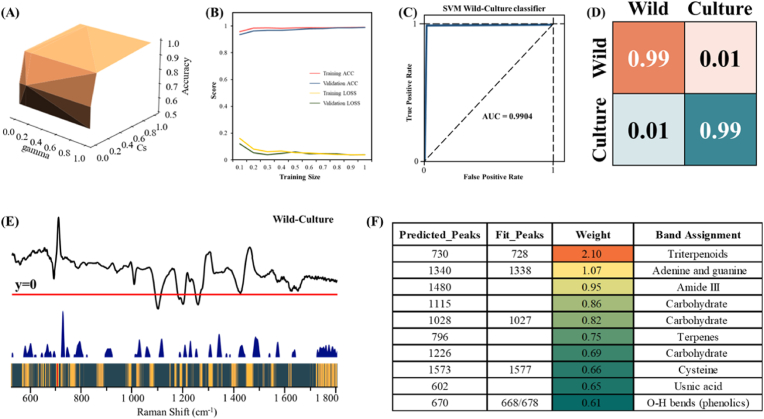
SVM performance evaluation and feature importance matching of metabolites. (A) SVM hyperparameter optimization. (B) Training curve. (C) ROC curve and AUC values. (D) Binary confusion matrix of SVM. (E) The spectral feature importance map found by SVM, where wavenumbers with high feature importance scores are important for cultivated and wild *O. sinensis* recognition. (F) Feature importance ranking and biological significance.

To better understand the decision-making process of the SVM, we further explain the classification results and examine the learned features, which provide important information for distinguishing between cultivated and wild *O. sinensis*. The optimal SVM model was trained using grid search (Fig. 4A) and learning curve (Fig. 4B) to ensure the stability of the model. The AUC value of the ROC curve was 0.9904 (Fig. 4C), and the confusion matrix showed an average prediction accuracy of 0.99 (Fig. 4D). These evaluation methods further support that the features learned by the SVM from the *O. sinensis* SERS signals are reliable. We assigned weight mappings based on the importance of Raman shifts in the spectral classification of cultivated and wild *O. sinensis*. The extracted spectral features are shown in Fig. 4E. It can be found that the important features correspond to the characteristic peaks of the difference curves of the two types of *O. sinensis*. These feature peaks can serve as crucial indicators for distinguishing between cultivated and wild *O. sinensis*. Furthermore, we ranked the importance of the spectral features. Fig. 4F shows the top ten features predicted by the SVM model. The results indicate that five of these feature peaks match those fitted from the raw spectrum, specifically at 728, 1338, 1027, 1577, and 668 cm^−1^. Additionally, two feature peaks align with the PCA loading, identified at 602 and 1480 cm^−1^. The remaining three characteristic peaks include molecular vibrations at 1115 and 1226 cm^−1^ produced by carbohydrates. As an energy source, all fungi require carbon-containing substances, and carbohydrates serve as a preferred carbon source (Singh et al., 2014), this substance suffers significant changes influenced by the growth environment and conditions (R. Tang et al., 2021). And the characteristic peak at 796 cm^−1^ is assigned to terpenes, which are abundant in *O. sinensis* and has anti-inflammatory, antibacterial and antioxidant effects (Qiu et al., 2020). The distribution of the above characteristic peaks indicates that, in making decisions, the SVM not only considers the significant peaks in the raw spectral distribution but also extracts information from other spectral bands to comprehensively determine the category of *O. sinensis*.

## 4 Discussions

*O. sinensis*, a well-known entomopathogenic fungus, is one of the most expensive edible and medicinal fungi in the world (Q.-H. Liu et al., 2023). However, due to its strict host-specificity and unique ecological characteristics (J. Zhang et al., 2023), wild *O. sinensis* is rare and difficult to obtain. Therefore, artificially cultivated *O. sinensis* with lower production costs and higher production output has emerged. Previous studies have shown that *O. sinensis* grown in different environments may undergo remarkable variations in their compositions (R. Tang et al., 2021). This has drawn attention to the differentiation between cultivated and wild *O. sinensis* and the detection of differences in their active components. In this study, we utilized SERS technology to achieve rapid measurement of chemical components in *O. sinensis*. Compared to existing methods for distinguishing cultivated and wild TCMs, such as chromatography, omics, and RNA sequencing (Sun et al., 2010), SERS is fast, reliable, and cost-effective (J.-W. Tang et al., 2022). This method has been applied to the identification of active components in various TCMs, such as *Coptis chinensis* and *Phellodendron amurense* (Zhao et al., 2014). Applying this method to detect *O. sinensis*, it was found that both types of data contain unique characteristic peaks, and the same peaks exhibit different intensities due to the influence of chemical composition content.

To further confirm the differences in biochemical components between cultivated and wild *O. sinensis*, we conducted a metabolomic analysis based on UPLC-Orbitrap-HRMS. The PCA and OPLS-DA analyses of SERS spectral data and MS data showed differences in metabolic profiles between wild and cultivated *O. sinensis*. Specifically, nine characteristic peaks with significant differences in Raman intensity were detected by SERS between wild and cultivated *O. sinensis*, which were mainly attributed to amino acids, nucleic acids, and carbohydrate components, and their contents were higher in cultivated *O. sinensis*. Among them, amino acids, carbohydrates, nucleosides, and nucleotides were consistently higher in wild *O. sinensis* than in cultivated *O. sinensis*. Amino acids are important components for maintaining homeostasis in the body and are key precursors for the synthesis of low molecular weight nitrogen-containing compounds (Wu, 2009). Previous studies have shown that *O. sinensis* contains all essential amino acids and some non-essential amino acids. In a comparative analysis, Chen et al. analyzed the amino acid composition of wild and cultivated *O. sinensis* and found that wild *O. sinensis* contained higher levels of total amino acids than cultivated *O. sinensis* (L. Chen et al., 2018; Wu, 2009). In this study, SERS detected that the characteristic peaks at 678 cm^−1^, 956 cm^−1^ and 1577 cm^−1^ belonging to amino acid components were more intense in wild *O. sinensis* than in cultivated *O. sinensis*. In addition, previous studies have shown that the average molecular weight of polysaccharides in *O. sinensis* is between 10^3^-10^6^ Da, mainly composed of glucose, mannitol, and galactose, which are the main active ingredients of *O. sinensis* (Yan et al., 2014). SERS detection showed that characteristic peaks at 808 cm^−1^, 855 cm^−1^, 1026 cm^−1^, and 1043 cm^−1^ were attributed to carbohydrates. Among them, Raman shift at 808 cm^−1^ and 1043 cm^−1^ had higher intensity in cultivated *O. sinensis*, while the SERS peak generated by *v* (C-C) of galactose and *v* (C-O) and *v* (C-C) of polysaccharides had a greater response in cultivated *O. sinensis*. Furthermore, nucleic acids are important compounds in O. sinensis and have multiple pharmacological effects. The characteristic peak at 1338 cm^−1^ is only found in wild *O. sinensis* and is attributed to the vibration of adenine and guanine. A higher nucleic acid component is essential for harsh growth environments and survival. Therefore, SERS detection can effectively obtain signals of the intensity of chemical component content in *O. sinensis* samples. This study next used UPLC-Orbitrap-HRMS to analyze the differential metabolites of wild and cultivated *O. sinensis* and found that the contents of amino acids, nucleic acids, and carbohydrate components in cultivated O. sinensis were higher, which was consistent with the validity of the SERS detection results. Despite SERS and UPLC-Orbitrap-HRMS providing detailed explanations for the differences in *O. sinensis* metabolites, advanced data analysis methods are still required for large-scale and rapid identification of wild and cultivated *O. sinensis* samples.

The combination of SERS technology and ML algorithms shows great potential in the analysis of components (Z. Li et al., 2024), sources (Ma et al., 2023), and quality (L. Zhang et al., 2023) of TCM. For example, Zhou et al. used DT, SVM, and Naive Bayes (NB) algorithms to achieve rapid, universal, and accurate identification of chemical composition changes in multi-component TCM. Their detection of three TCM decoctions achieved a classification accuracy of 97.78% (W. Zhou et al., 2024). In another study using SERS technology to detect TCM pharmacodynamic substances, a SERS substrate with high sensitivity and low detection limit was prepared to achieve quantitative detection of active ingredients in honeysuckle TCM (Yuan et al., 2024). Combined with three ML algorithms, the recognition accuracy reached 82%, providing effective support for chemical substance detection in TCM (Yuan et al., 2024). The results indicate a promising prospect for the combined application of SERS and ML in analyzing the chemical composition of TCM. Therefore, in this study, we employed various ML algorithms and different evaluation metrics for comprehensive analysis and assessment of *O. sinensis* SERS data. The identification accuracy of the optimal discriminative model SVM was 97.99%, with a 5-fold cross-validation score of 96.20%. However, despite the satisfactory results achieved by ML algorithms, the ML model methods are complex, and their internal mechanisms are difficult to understand. The relative importance of each variable is hard to estimate, which increases the uncertainty of ML in SERS applications. For spectroscopists and biologists, they are often interested in the mechanisms and factors responsible for modeling outputs rather than just precise modeling. To address this issue, we constructed feature importance maps for SVM. Based on the weight distribution of each Raman shift in the maps, we can gain insights into the key factors selected by SVM in distinguishing between cultivated and wild *O. sinensis* processes. Among the top ten characteristic peaks include triterpenoids (728 cm^−1^) which are abundant in *O. sinensis* (Wang et al., 2023a), guanine and adenine (1338 cm^−1^) exhibiting significant differences between cultivated and wild *O. sinensis* (J. Zhang et al., 2023), as well as carbohydrates (1115, 1208, and 1226 cm^−1^) which are significantly affected by the environment, climate, and geographical location (J. Zhang et al., 2023). The presence of these differing substances provides compelling evidence for the reliability of SVM.

Currently, the SERS technique has been studied for years as a method to analyze TCM components and quality, but it is still in its early stages (G.-L. Xu et al., 2014). There are several shortcomings in the existing research that are debatable. Firstly, the number of cultivated and wild *O. sinensis* samples included in this study is limited, and the existing SERS signals do not cover the complete data space of the sample population, making it challenging to obtain accurate and ideal models. Secondly, *O. sinensis* samples in each category come from different regions, and geographical and environmental differences may interfere with the efficiency of the model in learning the internal rules of similar samples. Therefore, we should collect sufficient samples from different times and spaces to enhance the model's generalization ability. Additionally, determining the preparation process of standardized and standardized SERS substrates and *O. sinensis* samples, as well as the process-based data processing methods, will greatly promote the application of SERS technology in TCM detection.

In summary, we have developed an intelligent identification method to distinguish between cultivated and wild *O. sinensis*, providing a theoretical basis for differentiating the metabolic differences between the two types and demonstrating the feasibility of combining SERS technology with ML algorithms in the field of TCM detection. The results of this study advocate integrating SERS with ML as a potent tool for the authentication and quality assessment of *O. sinensis*, facilitating the protection of consumers and legitimate businesses against fraudulent practices. This study not only enhances our understanding of the spectral properties of *O. sinensis* but also contributes to the broader field of quality control in TCM, providing a framework for future research aimed at the sustainable management and utilization of this valuable natural resource. Furthermore, this approach could offer a more convenient, economical, and faster solution to combat market fraud, making it an invaluable tool in ongoing efforts to ensure this essential medicinal resource's authenticity and therapeutic value.

## 5 Conclusion

This study employed a combination of the SERS technique and ML analysis to discriminate between wild and cultivated *O. sinensis*. The results showed significant differences in the characteristic peaks within the SERS metabolic profiles of the two types of *O. sinensis*. These differences were confirmed through the UPLC-Orbitrap-HRMS method, validating the composition and content variations obtained in the SERS spectra. To achieve intelligent identification of cultivated and wild *O. sinensis*, we explored the performance of various ML algorithms on the SERS data. The SVM algorithm achieved the best classification performance, with an accuracy of 97.99% and a 5-fold cross-validation score of 96.20%, indicating the applicability of the SVM algorithm in discriminating TCM samples. Furthermore, a feature importance map was utilized to interpret the role of SERS shifts in the SVM decision-making process. By assigning different weights to the characteristic peaks, we discovered that components such as triterpenoids, adenine, and carbohydrates play significant roles in ML classification. In summary, the combination of SERS technology and ML methods enables efficient and rapid identification of wild and cultivated *O. sinensis*. This work not only provides more comprehensive chemical analysis evidence for the quality assessment of *O. sinensis* but also lays a solid foundation for promoting the intelligent and automated transformation of TCM product identification.

## Credit author statement

LW conceived and designed the experiments. LW and YRT provided platforms and resources. LW contributed to project administration and student supervision. LW contributed to the funding acquisitions. QL, JT, ZM, YH, QY, and JC carried out the computational and experimental investigations. QL, JT, and LW wrote and revised the manuscript. All authors read, revised, and approved the final version of the manuscript.

## Funding statement

This study was financially supported by the 10.13039/501100021171Guangdong Basic and Applied Basic Research Foundation [Grant No. 2022A1515220023] and the Research Foundation for Advanced Talents of Guandong Provincial People's Hospital [Grant No. KY012023293]. The funders had no role in the study design, data collection, data analysis, interpretation, and manuscript writing.

## Declaration of competing interest

The authors declare that the research was conducted in the absence of any commercial or financial relationships that could be construed as a potential conflict of interest.

## Data Availability

Data will be made available on request.

## References

[bib1] Baral B. (2017). Entomopathogenicity and biological attributes of Himalayan treasured fungus *Ophiocordyceps sinensis* (Yarsagumba). Journal of Fungi.

[bib2] Chatnarin S., Thirabunyanon M. (2023). Potential bioactivities via anticancer, antioxidant, and immunomodulatory properties of cultured mycelial enriched β-D-glucan polysaccharides from a novel fungus *Ophiocordyceps sinensis* OS8. Front. Immunol..

[bib3] Chen H., Tan C., Li H. (2021). Discrimination between wild-grown and cultivated *Gastrodia elata* by near-infrared spectroscopy and chemometrics. Vib. Spectrosc..

[bib4] Chen L., Liu Y., Guo Q., Zheng Q., Zhang W. (2018). Metabolomic comparison between wild *Ophiocordyceps sinensis* and artificial cultured *Cordyceps militaris*. Biomed. Chromatogr..

[bib5] Du C., Zhou J., Liu J. (2017). Identification of Chinese medicinal fungus *Cordyceps sinensis* by depth-profiling mid-infrared photoacoustic spectroscopy. Spectrochim. Acta Mol. Biomol. Spectrosc..

[bib6] Fan Q., Ding H., Mo H., Tang Y., Wu G., Yin L. (2024). Cervical cancer biomarker screening based on Raman spectroscopy and multivariate statistical analysis. Spectrochim. Acta Mol. Biomol. Spectrosc..

[bib7] Grosch R., Guo L.-X., Xu X.-M., Liang F.-R., Yuan J.-P., Peng J., Wang J.-H. (2015). Morphological observations and fatty acid composition of indoor-cultivated *Cordyceps sinensis* at a high-altitude Laboratory on sejila mountain, Tibet. PLoS One.

[bib8] Hayat S., Hayat Q., Alyemeni M.N., Wani A.S., Pichtel J., Ahmad A. (2012). Role of proline under changing environments: a review. Plant Signal. Behav..

[bib9] He L., Xie F., Zhou G., Chen Z.H., Wang J.Y., Wang C.G. (2023). Transcriptome and metabonomics combined analysis revealed the energy supply mechanism involved in fruiting body initiation in Chinese *cordyceps*. Sci. Rep..

[bib10] Jang S.-H., Kim S.-H., Lee H.-Y., Jang S.-H., Jang H., Chae S.-W., Sin H.-S. (2015). Immune-modulating activity of extract prepared from mycelial culture of Chinese caterpillar mushroom, *Ophiocordyceps sinensis* (Ascomycetes). Int. J. Med. Mushrooms.

[bib11] Jędrejko K.J., Lazur J., Muszyńska B. (2021). *Cordyceps militaris*: an overview of its chemical constituents in relation to biological activity. Foods.

[bib12] Koh J.-H., Kim J.-M., Chang U.-J., Suh H.-J. (2003). Hypocholesterolemic effect of hot-water extract from mycelia of *Cordyceps sinensis*. Biol. Pharm. Bull..

[bib13] Li Y., Liu Y., Han X., Jin H., Ma S. (2019). Arsenic species in *Cordyceps sinensis* and its potential health risks. Front. Pharmacol..

[bib14] Li Z., Han X., Fu L., Shi G., Xu S., Wang M., Cui J. (2024). Machine learning-driven Ag/SiO2/Cu/rice leaf SERS platform for intelligent identification of pharmacodynamic substances. Microchem. J..

[bib15] Liu Q.-H., Zhang Y.-D., Ma Z.-W., Qian Z.-M., Jiang Z.-H., Zhang W., Wang L. (2023). Fractional extraction and structural characterization of glycogen particles from the whole cultivated caterpillar fungus *Ophiocordyceps sinensis*. Int. J. Biol. Macromol..

[bib16] Liu Y., Wang J., Wang W., Zhang H., Zhang X., Han C. (2015). The chemical constituents and pharmacological actions of *Cordyceps sinensis*. Evid. base Compl. Alternative Med..

[bib17] Ljungdahl P.O., Daignan-Fornier B. (2012). Regulation of amino acid, nucleotide, and phosphate metabolism in Saccharomyces cerevisiae. Genetics.

[bib18] Lo H.-C., Hsieh C., Lin F.-Y., Hsu T.-H. (2013). A systematic review of the mysterious caterpillar fungus Ophiocordyceps sinensis in DongChongXiaCao (冬蟲夏草 dōng chóng xià cǎo) and related bioactive ingredients. Journal of Traditional and Complementary Medicine.

[bib19] Lyu J.-W., Zhang X.D., Tang J.-W., Zhao Y.-H., Liu S.-L., Zhao Y., Chen X.-L. (2023). Rapid prediction of multidrug-resistant *Klebsiella pneumoniae* through deep learning analysis of SERS spectra. Microbiol. Spectr..

[bib20] Ma Z.-W., Tang J.-W., Liu Q.-H., Mou J.-Y., Qiao R., Du Y., Wang L. (2023). Identification of geographic origins of *Morus alba* Linn. through surfaced enhanced Raman spectrometry and machine learning algorithms. J. Biomol. Struct. Dyn..

[bib21] Marques J., Martin D., Amado A.M., Lysenko V., Osório N., Batista de Carvalho L.A., Moreira da Silva A. (2021). Novel insights into *Corema album* berries: vibrational profile and biological activity. Plants.

[bib22] Mou J.-Y., Usman M., Tang J.-W., Yuan Q., Ma Z.-W., Wen X.-R., Wang L. (2024). Pseudo-Siamese network combined with label-free Raman spectroscopy for the quantification of mixed trace amounts of antibiotics in human milk: a feasibility study. Food Chem. X.

[bib23] Ohta Y., Lee J.-B., Hayashi K., Fujita A., Park D.K., Hayashi T. (2007). In vivo anti-influenza virus activity of an immunomodulatory acidic polysaccharide isolated from *Cordyceps militaris* grown on germinated soybeans. J. Agric. Food Chem..

[bib24] Payne W.Z., Kurouski D. (2021). Raman spectroscopy enables phenotyping and assessment of nutrition values of plants: a review. Plant Methods.

[bib25] Pećinar I., Krstić D., Caruso G., Popović-Djordjević J.B. (2021). Rapid characterization of hypanthium and seed in wild and cultivated *rosehip*: application of Raman microscopy combined with multivariate analysis. R. Soc. Open Sci..

[bib26] Qiu X., Cao L., Han R. (2020). Analysis of volatile components in different *Ophiocordyceps sinensis* and insect host products. Molecules.

[bib27] Scalise M., Pochini L., Galluccio M., Indiveri C. (2016). Glutamine transport. From energy supply to sensing and beyond. Biochimica et Biophysica Acta (BBA)-Bioenergetics.

[bib28] Shrestha B., Zhang W., Zhang Y., Liu X. (2010). What is the Chinese caterpillar fungus *Ophiocordyceps sinensis* (Ophiocordycipitaceae)?. Mycology.

[bib29] Singh S., Ranjan S., Negi P.S., Arif M. (2014). Optimization of nutritional necessities for in vitro culture of *Ophiocordyceps sinensis*. Int. J. Sci. Res..

[bib30] Souza B.W., Cerqueira M.A., Bourbon A.I., Pinheiro A.C., Martins J.T., Teixeira J.A., Vicente A.A. (2012). Chemical characterization and antioxidant activity of sulfated polysaccharide from the red seaweed *Gracilaria birdiae*. Food Hydrocolloids.

[bib31] Sun S., Chen J., Zhou Q., Lu G., Chan K. (2010). Application of mid-infrared spectroscopy in the quality control of traditional Chinese medicines. Planta Med..

[bib32] Synytsya A., Bleha R., Skrynnikova A., Babayeva T., Čopíková J., Kvasnička F., Klouček P. (2023). Mid-infrared spectroscopic study of cultivating medicinal fungi *ganoderma*: composition, development, and strain variability of basidiocarps. Journal of Fungi.

[bib33] Tan L., Liu S., Li X., He J., He L., Li Y., Guo J. (2023). The large molecular weight polysaccharide from wild *cordyceps* and its antitumor activity on H22 tumor-bearing mice. Molecules.

[bib34] Tang J.-W., Li F., Liu X., Wang J.-T., Xiong X.-S., Lu X.-Y., Tay A.C.Y. (2024). Detection of *Helicobacter pylori* infection in human gastric fluid through surface-enhanced Raman spectroscopy coupled with machine learning algorithms. Lab. Invest..

[bib35] Tang J.-W., Li J.-Q., Yin X.-C., Xu W.-W., Pan Y.-C., Liu Q.-H., Wang L. (2022). Rapid discrimination of clinically important pathogens through machine learning analysis of surface enhanced Raman spectra. Front. Microbiol..

[bib36] Tang J.-W., Lyu J.-W., Lai J.-X., Zhang X.-D., Du Y.-G., Zhang X.-Q., Gu B. (2023). Determination of Shigella spp. via label-free SERS spectra coupled with deep learning. Microchem. J..

[bib37] Tang J.W., Yuan Q., Wen X.R., Usman M., Tay A.C.Y., Wang L. (2024). Label‐free surface‐enhanced Raman spectroscopy coupled with machine learning algorithms in pathogenic microbial identification: current trends, challenges, and perspectives. Interdisciplinary Medicine.

[bib38] Tang R., Qiu X.-H., Cao L., Long H.-L., Han R.-C. (2021). Stage-and rearing-dependent metabolomics profiling of *Ophiocordyceps sinensis* and its pipeline products. Insects.

[bib39] Tong C., Luo J., Xie C., Wei J., Pan G., Zhou Z., Li C. (2023). Characterization and biological activities of melanin from the medicinal fungi *Ophiocordyceps sinensis*. Int. J. Mol. Sci..

[bib40] Usman M., Tang J.-W., Li F., Lai J.-X., Liu Q.-H., Liu W., Wang L. (2023). Recent advances in surface enhanced Raman spectroscopy for bacterial pathogen identifications. J. Adv. Res..

[bib41] Vaverkova V., Vrana O., Adam V., Pekarek T., Jampilek J., Babula P. (2014). The study of naphthoquinones and their complexes with DNA by using Raman spectroscopy and surface enhanced Raman spectroscopy: new insight into interactions of DNA with plant secondary metabolites. BioMed Res. Int..

[bib42] Wang L., Tang J.-W., Li F., Usman M., Wu C.-Y., Liu Q.-H., Gu B. (2022). Identification of bacterial pathogens at genus and species levels through combination of Raman spectrometry and deep-learning algorithms. Microbiol. Spectr..

[bib43] Wang P., Sun H., Yang W., Fang Y. (2022). Optical methods for label-free detection of bacteria. Biosensors.

[bib44] Wang Y., Yang L.H., Tong L.L., Yuan L., Ren B., Guo D.S. (2023). Comparative metabolic profiling of mycelia, fermentation broth, spore powder and fruiting bodies of *Ophiocordyceps gracilis* by LC–MS/MS. Phytochem. Anal..

[bib45] Wang Y., Yang Z., Bao D., Li B., Yin X., Wu Y., Zou G. (2021). Improving hypoxia adaption causes distinct effects on growth and bioactive compounds synthesis in an entomopathogenic fungus *Cordyceps militaris*. Front. Microbiol..

[bib46] Wang Y., Yu C., Ji H., Liu Z., Wang X., Ji Y., Li Y. (2023). Label-free therapeutic drug monitoring in human serum by the 3-step surface enhanced Raman spectroscopy and multivariate analysis. Chem. Eng. J..

[bib47] Wang Y., Zhang B., Guo M., Wang C., Wang Q., Zhang L., Zhang Y. (2023). Rapid detection of cordycepin in food by surface-enhanced Raman technique. Journal of Future Foods.

[bib48] Wang Z., Ye J., Zhang K., Ding L., Granzier-Nakajima T., Ranasinghe J.C., Terrones M. (2022). Rapid biomarker screening of Alzheimer's disease by interpretable machine learning and graphene-assisted Raman spectroscopy. ACS Nano.

[bib49] Wei Y., Zhang L., Wang J., Wang W., Niyati N., Guo Y., Wang X. (2021). Chinese caterpillar fungus (*Ophiocordyceps sinensis*) in China: current distribution, trading, and futures under climate change and overexploitation. Sci. Total Environ..

[bib50] Wiercigroch E., Szafraniec E., Czamara K., Pacia M.Z., Majzner K., Kochan K., Malek K. (2017). Raman and infrared spectroscopy of carbohydrates: a review. Spectrochim. Acta Mol. Biomol. Spectrosc..

[bib51] Wu G. (2009). Amino acids: metabolism, functions, and nutrition. Amino acids.

[bib52] Xie M., Lu W., Gu S., Lu J., Wu H., Yao L., Wang Q. (2024). A rapid localization and analysis method for isoquinoline alkaloids with fluorescence in *Coptis chinensis* Franch. By fabricating the nano-silver sol as a substrate for surface-enhanced Raman spectroscopy. Anal. Chim. Acta.

[bib53] Xu C., Wu F., Zou Z., Mao L., Lin S. (2023). Discovery of the chemical constituents, structural characteristics, and pharmacological functions of Chinese caterpillar fungus. Open Chem..

[bib54] Xu C., Wu F., Zou Z., Mao L., Lin S. (2023). Discovery of the chemical constituents, structural characteristics, and pharmacological functions of Chinese caterpillar fungus. Open Chem..

[bib55] Xu G.-L., Xie M., Yang X.-Y., Song Y., Yan C., Yang Y., Wang Y. (2014). Spectrum-effect relationships as a systematic approach to traditional Chinese medicine research: current status and future perspectives. Molecules.

[bib56] Xu J., Huang Y., Chen X.X., Zheng S.C., Chen P., Mo M.H. (2016). The mechanisms of pharmacological activities of *Ophiocordyceps sinensis* fungi. Phytother Res..

[bib57] Xu Q., Zhao Z., Sun Y., Mackay R.P., Li Y.-Q. (2018). Extraction optimization for phenols and flavonoids from cultured mycelia of *cordyceps ophioglossoides* and exploration of bioactivities of its aqueous and ethanol extracts. Biomed. J..

[bib58] Xu Y.F., Létisse F., Absalan F., Lu W., Kuznetsova E., Brown G., Rabinowitz J.D. (2013). Nucleotide degradation and ribose salvage in yeast. Mol. Syst. Biol..

[bib59] Yan J.-K., Wang W.-Q., Wu J.-Y. (2014). Recent advances in *Cordyceps sinensis* polysaccharides: mycelial fermentation, isolation, structure, and bioactivities: a review. J. Funct.Foods.

[bib60] Yang C., Zhao Y., Jiang S., Sun X., Wang X., Wang Z., Li Y. (2024). A breakthrough in phytochemical profiling: ultra-sensitive surface-enhanced Raman spectroscopy platform for detecting bioactive components in medicinal and edible plants. Microchim. Acta.

[bib61] Yang C., Zhao Y., Jiang S., Sun X., Wang X., Wang Z., Li Y. (2024). A breakthrough in phytochemical profiling: ultra-sensitive surface-enhanced Raman spectroscopy platform for detecting bioactive components in medicinal and edible plants. Microchim. Acta.

[bib62] You Q., Li Q., Zheng H., Hu Z., Zhou Y., Wang B. (2017). Discerning silk produced by Bombyx mori from those produced by wild species using an enzyme-linked immunosorbent assay combined with conventional methods. J. Agric. Food Chem..

[bib63] Yuan W., Han X., Shi G., Wang M., Zhou W., Cui J., Wang L. (2024). Machine learning-driven multi-level composite SERS platform for trace detection of chlorogenic acid as pharmacodynamic substance in honeysuckle. Opt Laser. Technol..

[bib64] Yue K., Ye M., Lin X., Zhou Z. (2013). The artificial cultivation of medicinal caterpillar fungus, *Ophiocordyceps sinensis* (Ascomycetes): a review. Int. J. Med. Mushrooms.

[bib65] Zhang B., Li B., Men X.-H., Xu Z.-W., Wu H., Qin X.-T., Jin L.-Q. (2020). Proteome sequencing and analysis of *Ophiocordyceps sinensis* at different culture periods. BMC Genom..

[bib66] Zhang J., Wang N., Chen W., Zhang W., Zhang H., Yu H., Yi Y. (2023). Integrated metabolomics and transcriptomics reveal metabolites difference between wild and cultivated *Ophiocordyceps sinensis*. Food Res. Int..

[bib67] Zhang J., Zhong X., Li S., Zhang G., Liu X. (2015). Metabolic characterization of natural and cultured *Ophicordyceps sinensis* from different origins by 1H NMR spectroscopy. J. Pharmaceut. Biomed. Anal..

[bib68] Zhang L.-Y., Tian B., Huang Y.-H., Gu B., Ju P., Luo Y., Wang L. (2023). Classification and prediction of *Klebsiella pneumoniae* strains with different MLST allelic profiles via SERS spectral analysis. PeerJ.

[bib69] Zhang L., Zhang C., Li W., Li L., Zhang P., Zhu C., Sun H. (2023). Rapid indentification of auramine O dyeing adulteration in dendrobium officinale, saffron and curcuma by SERS Raman spectroscopy combined with SSA-BP neural networks model. Foods.

[bib70] Zhao J., Liu Y., Fales A.M., Register J., Yuan H., Vo‐Dinh T. (2014). Direct analysis of traditional Chinese medicines using surface‐enhanced Raman scattering (SERS). Drug Test. Anal..

[bib71] Zhou W., Han X., Wu Y., Shi G., Xu S., Wang M., Li Z. (2024). High-performance grating-like SERS substrate based on machine learning for ultrasensitive detection of Zexie-Baizhu decoction. Heliyon.

[bib72] Zhou X.-W., Li L.-J., Tian E.-W. (2014). Advances in research of the artificial cultivation of *Ophiocordyceps sinensis* in China. Crit. Rev. Biotechnol..

[bib73] Zhu G., Zhu X., Fan Q., Wan X. (2011). Raman spectra of amino acids and their aqueous solutions. Spectrochim. Acta Mol. Biomol. Spectrosc..

[bib74] Zuo H.-L., Chen S.-J., Zhang D.-L., Zhao J., Yang F.-Q., Xia Z.-N. (2013). Quality evaluation of natural *Cordyceps sinensis* from different collecting places in China by the contents of nucleosides and heavy metals. Anal. Methods.

