# Supplementary material for: Rapid discrimination between wild and cultivated Ophiocordyceps sinensis through comparative analysis of label-free SERS technique and mass spectrometry
Source: Curr Res Food Sci. 2024 Aug 14;9:100820. doi: 10.1016/j.crfs.2024.100820 (PMC11387260; doi:10.1016/j.crfs.2024.100820)
Supplement: Multimedia component 1 [file mmc1.docx]

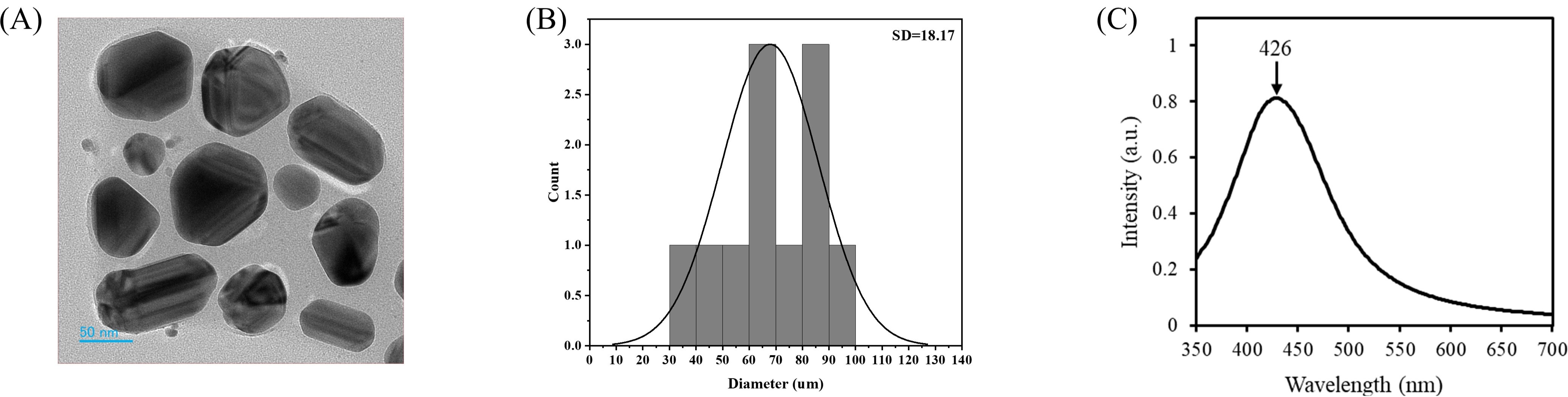


**Supplementary Figure S1** Structural characteristics of the silver nanoparticles used in this study. (A) Transmission electron microscopy (TEM) image of AgNPs. (B) Particle size distribution of AgNPs. (C) Ultraviolet-visible (UV-Vis) absorbance spectrum of AgNPs. Note: we acquired transmission electron microscope (TEM) images of AgNPs. The particle size distribution of the silver nanoparticles was analyzed using the image processing software ImageJ (NIH, USA, Java 1.8.0_345 64-bit), revealing sizes predominantly within the range of 30-100 nm. Ultraviolet-visible (UV-Vis) spectroscopy of the AgNPs solution indicated good stability of the prepared base solution.
