# Supplementary material for: Rapid discrimination between wild and cultivated Ophiocordyceps sinensis through comparative analysis of label-free SERS technique and mass spectrometry
Source: Curr Res Food Sci. 2024 Aug 14;9:100820. doi: 10.1016/j.crfs.2024.100820 (PMC11387260; doi:10.1016/j.crfs.2024.100820)
Supplement: Multimedia component 2 [file mmc2.docx]

**Supplementary Table S1** Parameter setting of machine learning models for the SERS signals of wild and cultivated *O. sinensis*.

| **Algorithms** | **Parameter Range** | **Optimum Parameter** |
| --- | --- | --- |
| **AdaBoost** | **learning_rate** = [0.1, 1, 0.01, 0.001],  **n_estimators** = [50, 60, 70, 80, 90, 100, 110, 120, 130, 140, 150, 160, 170, 180] | **learning_rate** = 1, **n_estimators** = 180 |
| **Decision Tree** | **criterion** = ['gini', 'entropy'],  **max_depth** = range (1, 30), **max_features** = [21, 22, 23, 24, 25, 26, 28, 29, 30, 'auto'] | **criterion** = ' entropy',  **max_depth** = 19,  **max_features** = 33 |
| **GBoost** | **n_estimators =** [120, 130, 140, 145, 150, 160, 170, 175, 180, 185],  **learning_r =** [0.1, 1, 0.01, 0.001] | **learning_rate** = 0.1, **n_estimators** = 70 |
| **Random Forest** | **Criterion** = ['gini', 'entropy'],  **max_depth** = range (1, 10),  **n_estimators** = [50, 60, 70, 80, 90, 100, 110, 120, 130, 140, 150, 160, 170, 180] | **criterion** = 'entropy', **max_depth** = 25, **n_estimators** = 150 |
| **SVM** | **Cs =** [0.0001, 0.001, 0.01, 0.1, 1, 2, 3, 4, 5, 10],  **gamma =** [0.0001, 0.001, 0.01, 0.1, 1],  kernel = ['rbf', ' linear '] | **Cs =** 1,  **gamma =** 0.0001,  **kernel =** 'linear',  **probability =** True |
| **XGBoost** | **n_estimators =** [50, 60, 70, 80, 90, 100, 110, 120, 130, 140, 150, 160, 170, 180],  **learning_r =** [0.1, 1, 0.01, 0.001] | **learning_rate** = 1, **n_estimators** = 60 |
