# Supplementary material for: Rapid discrimination between wild and cultivated Ophiocordyceps sinensis through comparative analysis of label-free SERS technique and mass spectrometry
Source: Curr Res Food Sci. 2024 Aug 14;9:100820. doi: 10.1016/j.crfs.2024.100820 (PMC11387260; doi:10.1016/j.crfs.2024.100820)
Supplement: Multimedia component 3 [file mmc3.docx]

**Supplementary Table S2** Characteristic peaks of SERS spectra corresponding to the wild and cultivated *O. sinensis*.

| **Wavenumber (cm^-1^)** | **Wild** | **Culture** | **Band Assignment** | ***Ref.*** |
| --- | --- | --- | --- | --- |
| 648 |  |  | Flavonoids, flavone A-ring | (Bock, Felhofer, Mayer, & Gierlinger, 2021) |
| 668 |  |  | O-H bends (phenolics) | (Suriyakalaa et al., 2013) |
| 678 |  |  | Stretching frequency of C-S bond (Cysteine) | (Zhu, Zhu, Fan, & Wan, 2011) |
| 713 |  |  | CH_2_ oscillating vibration | (Weng et al., 2018) |
| 728 |  |  | Triterpenoids | (Heredia-Guerrero et al., 2014) |
| 808 |  |  | ν (CC), β (CCH) of 2-deoxy-D-ribose | (Wiercigroch et al., 2017) |
| 855 |  |  | ν (C-C) of galactose | (Souza et al., 2012) |
| 956 |  |  | Valine, Proline | (Fan, Ding, Mo, Tang, Wu, & Yin, 2024) |
| 1026 |  |  | *v* (C-O) and *v* (C-C) of polysaccharides | (Marques et al., 2021) |
| 1043 |  |  | β-D-glucan | (Synytsya et al., 2023) |
| 1338 |  |  | Adenine and guanine | (Vaverkova, Vrana, Adam, Pekarek, Jampilek, & Babula, 2014) |
| 1131/1133 |  |  | Ring vibration of adenine | (C. Muntean, Leopold, Halmagyi, & Valimareanu, 2013) |
| 1215/1216 |  |  | Xylan | (Efremov, Ariese, & Gooijer, 2008) |
| 1390 |  |  | Carotene | (Wang, Liao, Meng, Jiao, Huang, & Liu, 2019) |
| 1315/1312 |  |  | Flavones/chalcones | (Bock et al., 2021) |
| 1444/1445 |  |  | CH_2_ scissor of deoxyribose | (C. M. Muntean, Leopold, Tripon, Coste, & Halmagyi, 2015) |
| 1577/1578 |  |  | Cysteine | (Piot, Autran, & Manfait, 2000) |
| 1634/1635 |  |  | Flavone line attributed to C-O and C_2_-C_3_ stretching | (Teslova et al., 2007) |
| 1654 |  |  | Carbonyl | (Baranska, Roman, Cz Dobrowolski, Schulz, & Baranski, 2013) |

**Reference**

Baranska, M., Roman, M., Cz Dobrowolski, J., Schulz, H., & Baranski, R. (2013). Recent advances in Raman analysis of plants: alkaloids, carotenoids, and polyacetylenes. *Current Analytical Chemistry, 9*(1), 108-127.

Bock, P., Felhofer, M., Mayer, K., & Gierlinger, N. (2021). A guide to elucidate the hidden multicomponent layered structure of plant cuticles by Raman imaging. *Frontiers in Plant Science, 12*, 793330.

Efremov, E. V., Ariese, F., & Gooijer, C. (2008). Achievements in resonance Raman spectroscopy: Review of a technique with a distinct analytical chemistry potential. *Analytica chimica acta, 606*(2), 119-134.

Fan, Q., Ding, H., Mo, H., Tang, Y., Wu, G., & Yin, L. (2024). Cervical cancer biomarker screening based on Raman spectroscopy and multivariate statistical analysis. *Spectrochimica Acta Part A: Molecular and Biomolecular Spectroscopy, 317*, 124402.

Heredia-Guerrero, J. A., Benítez, J. J., Domínguez, E., Bayer, I. S., Cingolani, R., Athanassiou, A., & Heredia, A. (2014). Infrared and Raman spectroscopic features of plant cuticles: a review. *Frontiers in Plant Science, 5*, 305.

Marques, J., Martin, D., Amado, A. M., Lysenko, V., Osório, N., Batista de Carvalho, L. A., . . . Moreira da Silva, A. (2021). Novel insights into Corema album berries: Vibrational profile and biological activity. *Plants, 10*(9), 1761.

Muntean, C., Leopold, N., Halmagyi, A., & Valimareanu, S. (2013). Surface‐enhanced Raman scattering assessment of DNA from leaf tissues adsorbed on silver colloidal nanoparticles. *Journal of Raman Spectroscopy, 44*(6), 817-822.

Muntean, C. M., Leopold, N., Tripon, C., Coste, A., & Halmagyi, A. (2015). Surface-enhanced Raman spectroscopy of genomic DNA from in vitro grown tomato (Lycopersicon esculentum Mill.) cultivars before and after plant cryopreservation. *Spectrochimica Acta Part A: Molecular and Biomolecular Spectroscopy, 144*, 107-114.

Piot, O., Autran, J.-C., & Manfait, M. (2000). Spatial distribution of protein and phenolic constituents in wheat grain as probed by confocal Raman microspectroscopy. *Journal of Cereal Science, 32*(1), 57-71.

Souza, B. W., Cerqueira, M. A., Bourbon, A. I., Pinheiro, A. C., Martins, J. T., Teixeira, J. A., . . . Vicente, A. A. (2012). Chemical characterization and antioxidant activity of sulfated polysaccharide from the red seaweed Gracilaria birdiae. *Food Hydrocolloids, 27*(2), 287-292.

Suriyakalaa, U., Antony, J. J., Suganya, S., Siva, D., Sukirtha, R., Kamalakkannan, S., . . . Achiraman, S. (2013). Hepatocurative activity of biosynthesized silver nanoparticles fabricated using Andrographis paniculata. *Colloids and Surfaces B: Biointerfaces, 102*, 189-194.

Synytsya, A., Bleha, R., Skrynnikova, A., Babayeva, T., Čopíková, J., Kvasnička, F., . . . Klouček, P. (2023). Mid-Infrared Spectroscopic Study of Cultivating Medicinal Fungi Ganoderma: Composition, Development, and Strain Variability of Basidiocarps. *Journal of Fungi, 10*(1), 23.

Teslova, T., Corredor, C., Livingstone, R., Spataru, T., Birke, R. L., Lombardi, J. R., . . . Leona, M. (2007). Raman and surface‐enhanced Raman spectra of flavone and several hydroxy derivatives. *Journal of Raman Spectroscopy: An International Journal for Original Work in all Aspects of Raman Spectroscopy, Including Higher Order Processes, and also Brillouin and Rayleigh Scattering, 38*(7), 802-818.

Vaverkova, V., Vrana, O., Adam, V., Pekarek, T., Jampilek, J., & Babula, P. (2014). The study of naphthoquinones and their complexes with DNA by using Raman spectroscopy and surface enhanced Raman spectroscopy: new insight into interactions of DNA with plant secondary metabolites. *BioMed Research International, 2014*.

Wang, K., Liao, Y., Meng, Y., Jiao, X., Huang, W., & Liu, T. C.-y. (2019). The early, rapid, and non-destructive detection of citrus Huanglongbing (HLB) based on microscopic confocal Raman. *Food Analytical Methods, 12*, 2500-2508.

Weng, S., Qiu, M., Dong, R., Wang, F., Zhao, J., Huang, L., & Zhang, D. (2018). Quantitative determination of chlormequat chloride residue in wheat using surface-enhanced Raman spectroscopy. *International Journal of Analytical Chemistry, 2018*.

Wiercigroch, E., Szafraniec, E., Czamara, K., Pacia, M. Z., Majzner, K., Kochan, K., . . . Malek, K. (2017). Raman and infrared spectroscopy of carbohydrates: A review. *Spectrochimica Acta Part A: Molecular and Biomolecular Spectroscopy, 185*, 317-335.

Zhu, G., Zhu, X., Fan, Q., & Wan, X. (2011). Raman spectra of amino acids and their aqueous solutions. *Spectrochimica Acta Part A: Molecular and Biomolecular Spectroscopy, 78*(3), 1187-1195.
